# Transdifferentiation of MALME-3M and MCF-7 Cells toward Adipocyte-like Cells is Dependent on Clathrin-mediated Endocytosis

**DOI:** 10.1186/2193-1801-1-44

**Published:** 2012-10-30

**Authors:** Jaime Carcel-Trullols, Cristóbal Aguilar-Gallardo, Fernando Garcia-Alcalde, Miguel Angel Pardo-Cea, Joaquin Dopazo, Ana Conesa, Carlos Simón

**Affiliations:** Bioinformatics and Genomics Department, Prince Felipe Research Centre (CIPF), Avda. Autopista del Saler, 16-3 46012 Valencia, Spain

**Keywords:** Cell Transdifferentiation, Unsaturated Fatty Acids, PPARG, Perilipin 2, Loss of Pigmentation, Adipogenic Gene Markers, Clathrin, Clathrin-mediated Endocytosis

## Abstract

**Abstract:**

Enforced cell transdifferentiation of human cancer cells is a promising alternative to conventional chemotherapy. We previously identified albumin-associated lipid- and, more specifically, saturated fatty acid-induced transdifferentiation programs in human cancer cells (HCCLs). In this study, we further characterized the adipocyte-like cells, resulting from the transdifferentiation of human cancer cell lines MCF-7 and MALME-3M, and proposed a common mechanistic approach for these transdifferentiating programs. We showed the loss of pigmentation in MALME-3M cells treated with albumin-associated lipids, based on electron microscopic analysis, and the overexpression of perilipin 2 (PLIN2) by western blotting in MALME-3M and MCF-7 cells treated with unsaturated fatty acids. Comparing the gene expression profiles of naive melanoma MALME-3M cells and albumin-associated lipid-treated cells, based on RNA sequencing, we confirmed the transcriptional upregulation of some key adipogenic gene markers and also an alternative splicing of the adipogenic master regulator PPARG, that is probably related to the reported up regulated expression of the protein. Most importantly, these results also showed the upregulation of genes responsible for Clathrin (CLTC) and other adaptor-related proteins. An increase in CLTC expression in the transdifferentiated cells was confirmed by western blotting. Inactivation of CLTC by chlorpromazine (CHP), an inhibitor of CTLC mediated endocytosis (CME), and gene silencing by siRNAs, partially reversed the accumulation of neutral lipids observed in the transdifferentiated cells. These findings give a deeper insight into the phenotypic changes observed in HCCL to adipocyte-like transdifferentiation and point towards CME as a key pathway in distinct transdifferentiation programs.

**Disclosures:**

Simon C and Aguilar-Gallardo C are co-inventors of the International Patent Application No. PCT/EP2011/004941 entitled “Methods for tumor treatment and adipogenesis differentiation”.

**Electronic supplementary material:**

The online version of this article (doi:10.1186/2193-1801-1-44) contains supplementary material, which is available to authorized users.

## Introduction

Transdifferentiation involves reprogramming one type of adult cell into another mature cell type, without having to generate intermediary stem cells. It has received significant attention because it may have a considerable number of applications in cell and cancer therapy. Successful transdifferentiation from one cell type to another by overexpressing lineage-specific genes *in vivo* (Takeuchi & [Bruneau 2009]; Zhou et al. [2008]) and *in vitro* (Graf & [Enver 2009]; Ieda et al. [2010]) has been previously reported. Not only can cell transdifferentiation be enforced through the overexpression of the appropriate set of genes, but also through the application of specific molecules, which is a promising alternative to conventional chemotherapy for cancer treatment, for example, the use of *all-trans*-Retinoid acid (ATRA) for the treatment of acute promyelocitic leukemia (APL) (Kakizuka et al. [1991]). The fact that there was no treatment based on cell differentiation therapy for solid tumors prompted us to study whether novel transdifferentiating molecules secreted by human embryonic stem cells (hESCs) could prevent cancer progression. Our studies showed that, irrespective of the presence of hESCs, albumin-associated lipids (albuMAX®), and specifically poly- and monounsaturated fatty acids (linoleic, oleic, petroselinic and palmitoleic acids) accounted for the transdifferentiation of several distinct human cancer cell lines (HCCLs) into adipocyte-like cells (Ruiz-Vela et al. [2011]).

Endocytosis regulates the entry of nutrients, hormones and signaling factors into the cell, and serves to regulate the internalization of transmembrane receptors. During endocytosis the plasma membrane invaginates to form a new intracellular vesicle containing various cargo molecules and material to be internalized from the external environment (Conner & [Schmid 2003]). Clathrin (CLTC)-mediated endocytosis (CME) is the major endocytic route and is assumed to regulate ligand mediated signal transduction by disrupting ligand-receptor interactions after their uptake into endosomal compartments ( [Andersson 2012]). It can sequester the receptors in intracellular compartments, target ligand-receptor complexes to lysosomes for degradation, or recycle them by sending them back to the cell surface for re-use (Maxfield & Mc [Graw 2004]). Several *in vitro* studies have reported evidence for a relationship between the endocytosis of ligands such as Bone morphogenetic protein 2 (BMP2) and oxidized Low-density lipoprotein (ox-LDL), bound to their respective receptors, and their key roles in different transdifferentiation models (Rauch et al. [2002]; Yu et al. [2010]). Others have shown that the incorporation of insulin regulated glucose transporter 4 (GLUT4) into the cell plasma membrane, a process required for adipogenesis, is dependent on clathrin coated vesicles (Huang et al. [2007]). However, the existence of a direct correlation between endocytic ligand-receptor complex internalization and the transcription of genes controlling critical physiological events, such as cell transdifferentiation or adipogenesis, remains largely uninvestigated.

Our previous report identified similar transdifferentiation programs in HCCLS of completely different origins such as in cells from ovarian carcinoma, hepatocarcinoma, breast adenocarcinoma and melanoma (Ruiz-Vela et al. [2011]). In this study we aimed to characterize the adipocyte-like cells resulting from melanoma MALME-3M and breast carcinoma MCF-7 transdifferentiation programs and to set the key pathways that were concomitantly affected in these programs. In order to document induced-transdifferentiation we report the loss of pigmentation in MALME-3M cells, the upregulation of PLIN2 protein expression in the studied HCCLs and the upregulation of gene expression of PLIN2 and other commonly considered key adipogenic markers such as lipoprotein lipase (LPL) and peroxisome proliferator-activated receptor alpha (PPARA) in MALME-3M cells. These results also revealed an alternative gene splicing of the adipogenic master regulator PPARG that was likely to be related to the upregulation of the protein found in transdifferentiated MALME-3M cells, previously reported by our group (Ruiz-Vela et al. [2011]). Most importantly, our results also showed an increase in Clathrin (CLTC) expression and we provided evidence that Clathrin-mediated Endocytosis (CME) was essential for the transdifferentiation programs of the breast adenocarcinoma and melanoma HCCLs we investigated into adipocyte-like cells.

## Materials and methods

### Cell culture and transfection

MALME-3M melanoma (ATTC# HTB-64) and MCF-7 breast adenocarcinoma (ATTC#HTB-22) cells were cultured in growth media containing RPMI-1640, 10% fetal bovine serum (FBS) and 2 mM glutamine, following a standard 3 T3 protocol (Todaro & [Green 1963]). Albumin-associated lipids were obtained by adding GIBCO^TM^ AlbuMAX II from Invitrogen at a concentration of 1.6% w/v in RPMI media containing 10% FBS. To induce cell transdifferentiation, cells were cultured with albumin-associated lipids, whereas mock-treated cells were cultured in RPMI containing 10% FBS. After 24 hours cells were trypsinized, collected in plastic tubes and centrifuged at 5000 rpm.

CLTC-targeted siRNA and scrambled siRNA (control siRNA) were obtained from Dharmacon (Dharmacon RNA i Technologies). Sequences from CLTC-targeted siRNA and control siRNA were commercially provided. Cells were seeded according to the manufacturer’s protocol in serum-containing media without antibiotics and transfected with either 50 nM siRNA or 50 nM control siRNA with DharmaFECT (Dharmacon RNA i Technologies) in antibiotic-free media for 72 h.

### Gene expression analysis

Whole genome transcriptional profiles of both control MALME-3M cells and transdifferentiated cells, after 24 hours incubation with albumin-associated lipids, were obtained by RNA-seq. Briefly, total RNA was purified from cell cultures using Quick-RNA MiniPrep^TM^ (Zymo Research) protocol and strand-specific 38 nt pair-end Solexa libraries were prepared following the dUTP method (Parkhomchuk et al. [2009]; Levin et al. [2010]).

Sequencing reads were mapped to the human genome (GRCh37/hg19 assembly) using Tophat 1.1.4 with standard parameters and indicating the corresponding mate inner distance (46 bp for 0 h and 44 bp for 24 h) (Trapnell et al. [2009]). Gene counts were estimated using htseq-count ( http://www-huber.embl.de/users/anders/HTSeq) with standard parameters, using the human annotations obtained from Ensembl 60. Differentially expressed genes between MALME-3M and adipose cell types were estimated by means of the NOISeq non parametrical statistical method (Tarazona et al. [2011]) and enrichment of functional categories was calculated by the GOSeq method (Young et al. [2010]) an adaptation of the Fisher’s Exact test to sequencing data. To assess gene differential expression three technical replicates per sample were analyzed with a total sequencing depth of 300 milions of reads per sample.

### Antibodies

Polyclonal anti-Actin, anti-CLTC and anti-ADFP (anti PLIN2) antibodies were purchased from Abcam® (Cambridge).

### Western blots

For immunoblot analysis, cell extracts were prepared in RIPA buffer (Sigma-Aldrich®), containing 1% SDS. Proteins from the various lysates were separated by SDS/PAGE in 4-12% Bis-Tris gels and transferred to PVDF membranes (GE Healthcare, Amersham Biosciences). Membranes were incubated with the corresponding primary antibodies and with appropriate secondary antibodies. Proteins were visualized by standard chemiluminescence (GE Healthcare), and the bands were quantified by densitometric analysis with an Image Quant ECL (GE Healthcare). The ratios between the bands of interest and the corresponding actin bands were plotted.

### Electron microscopy

MALME 3M cells and transdifferentiated cells, after 24 hours incubation with albumin-associated lipids, were seeded on Permanox slides (Nalge Nunc International, Naperville, IL) at a density of 2000 cells x 4,2 cm^2^/well. Cells were fixed with 3.5% glutaraldehyde (1 h, 37°C), treated with 2% osmium tetroxide (1 h, RT) and stained with 2% uranyl acetate away from light (2 h, 4°C). Cells were rinsed in sodium phosphate buffer (0.1 M), dehydrated in ethanol, and infiltrated overnight in araldite resins (Durcupan, Fluka, Buchs SG, Switzerland). After polymerization, embedded cultures were detached from the chamber slide and glued to Araldite blocks. Semi-thin sections (1.5 μm) were cut with an Ultracut UC-6 (Leica, Heidelberg, Germany), mounted onto slides and stained with 1% toluidine blue. Semi-thin sections were glued to araldite blocks and detached from the glass slide by freezing-thawing cycles using liquid nitrogen. Ultrathin sections (0.07 μm) were prepared with an Ultracut and then stained with lead citrate. Finally, photomicrographs were obtained under an FEI transmission electron microscope (Tecnai Spirit G2) using a digital camera (Soft Imaging System, Olympus).

### Nile Red staining

Neutral lipid quantification was performed with Nile-Red (Greenspan et al. [1985]). Cells (10^5^) were washed twice in PBS and then incubated with 25 μg/ml of Nile Red (Invitrogen) in PBS (30 min, 4°C). After incubation, cells were filtered through a 30 μm sieve (CellTrics, Partec). Fluorescent emission (525 nm) was registered in the FL2 channel by a Beckman Coulter Flow Cytometer (Cytomics™ FC 500) and data files were analyzed using the FlowJo flow cytometer analysis software.

### Oil Red O staining

Lipid droplets (LDs) were stained with Oil Red O (Kutt & [Tsaltas 1959]). A stock of Oil Red O solution was prepared in 2-propanol (0.3%), vortexed and filtered through a 0.8 μm sieve before staining. The cells (10^5^) grown on 6-well plates were washed twice in PBS and fixed with 4% of formaldehyde (Panreac) for 30 min. After fixation, cells were washed 3 times in PBS and stained with Oil Red O (60 volumes of stock solution: 40 volumes of water). Cells were washed with cold PBS (at least 3 times) and then stained in filtered hematoxylin (Sigma-Aldrich) for 2 min. After staining, cells were washed with PBS and pictures were taken under a phase contrast microscope (Leica DMIL) using the Leica Application Suite version 2.4.0 R1 software (Leica Microsystems, Switzerland).

## Results

### Early transdifferentiation markers: albumin-associated lipids induce a loss of pigmentation in MALME-3M cells

**Figure 1:**
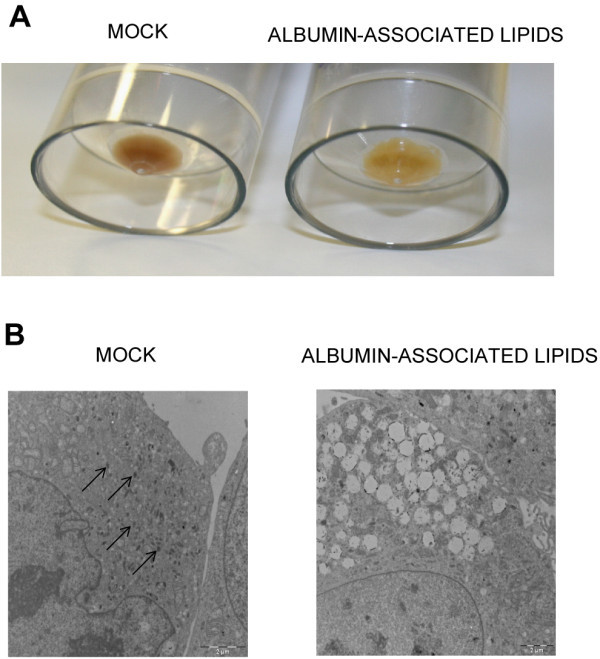
**Loss of pigmentation in albumin-associated lipid-treated MALME-3M cells.** MALME-3M cells were mock-treated or treated with albumin-associated lipids for 24 hours (**A**). Electron Microscopy of melanosome pigmentation in MALME-3M cells mock-treated or treated with Albumin-associated lipids for 24 hours (**B**). The arrows indicate the dark vesicles that are hallmarks of melanosomes and disappear upon albumin-associated lipid treatment.

We first checked changes in pigmentation in MALME-3M cells, as such changes could suggest the initiation of a transdifferentiation process. As Figure 1A shows, the pellet from cells treated with albumin-associated lipids was much lighter in color than the pellet corresponding to the mock-treated cells, indicating a marked loss of pigmentation in the albumin-associated lipid-treated cells. In order to analyze the ultrastructural features responsible for the observed loss of pigmentation in the treated cells we used Electron Microscopy (EM). EM studies showed large clusters of dark vesicles in mock-treated MALME-3M cells that were not present in the albumin-associated lipid-treated cells (Figure 1B and Additional file 1: Supporting information C at higher magnification). These vesicles are hallmarks of melanosomes and are responsible for the storage of melanin pigments (Raposo & [Marks 2007]). Thus, the EM studies suggested that the loss of pigmentation was due to impaired melanosome biogenesis.

Melan-A (MLANA) gene expression is restricted to melanocytes, melanomas, and retinal pigment epithelium, and is directly implicated in melanosome biogenesis (De Maziere et al. [2002]). RNA-seq data indicated that this key melanocytic marker was downregulated with a 1.41 fold-change in the 24-hour period analyzed.

### Late Adipocyte transdifferentiation markers perilipin 2 (PLIN2), lipoprotein lipase (LPL), and peroxisome proliferator-activated receptor alpha (PPARA) are increased in transdifferentiated MALME-3M and MCF-7 cells

To confirm that MALME-3M cells were effectively transdifferentiated into adipocyte-like cells, we checked the presence of hallmarks of adipocyte transdifferentiation. PLIN2 is a lipid storage droplet-associated protein that localizes at the surface of neutral lipid droplets (LDs) in adipocytes (Brasaemle et al. [1997]; Heid et al. [1998]). RNA-seq data showed that, compared to control cells, PLIN2 gene expression was upregulated by a fold change of 2.08 in albumin-associated lipid-treated cells over 96 hours. Other adipocyte marker genes such as lipoprotein lipase (LPL) and peroxisome proliferator-activated receptor alpha (PPARA) were also upregulated by a fold change of 1.71 and 1.30, respectively.

**Figure 2:**
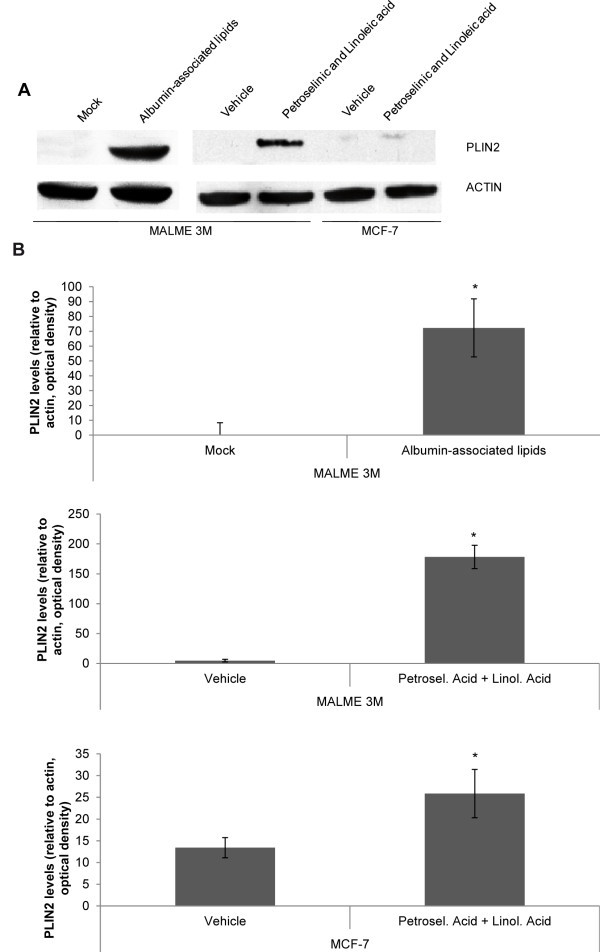
**PLIN2 expression is increased in the cells treated with albumin-associated lipids, petroselinic and linoleic acids.** MALME-3M and MCF-7 cells were treated with albumin-associated lipids, petroselinic acid (100 μg/ml) and linoleic acid (100 μg/ml) for 96 hours. The western blot band intensity corresponding to PLIN2 was normalized to the corresponding Actin band (**A**) for each sample (**B**). The average values from densitometry analyses of three separate experiments are shown. *P <0.05 compared to each control.

Western blot analysis of PLIN2 showed that the protein was absent in the MALME-3M mock-treated cells, and in cells incubated with vehicles but it was markedly expressed in the cells incubated for 96 hours with albumin-associated lipids or with a mixture of linoleic and petroselinic acids (100 μg/ml of each) (Figure 2A). In MCF-7 cells there was also a significant increase in PLIN2 expression in linoleic and petroselinic treated cells (25.87±5.56) versus vehicle treated cells (13.42 ± 2.33) (Figure 2B).

### Deep sequencing analysis of the transdifferentiation process to adipocyte-like cells demonstrates the implication of genes that participate in endocytosis

In order to gain insight into the mechanisms that albumin-associated lipid enriched media acts as a transdifferentiating media for human cancer cell lines (HCCLs) (Ruiz-Vela et al. [2011]; Ruiz-Vela et al. [2009]), we examined how gene expression in MALME-3M cells is regulated during the transdifferentiation process, using deep sequencing. We cultured MALME-3M cells with RPMI medium in the presence of albumin-associated lipids for 24 hours. As negative controls MALME-3M cells were cultured with RPMI-1640 containing 10% fetal bovine serum (RPMI + 10% FBS) for the same period of time. mRNA was extracted and gene expression was quantified by transcriptome sequencing (RNA-seq). Over 2,000 genes were identified as differentially expressed between the treated and the untreated cells. Gene expression analysis of the MALME-3M to adipocyte-like transdifferentiation demonstrated a significant upregulation of genes that participate in functions related to endocytosis (Table 1A and Additional file 2: Supporting information A). Functional enrichment analysis of differentially expressed genes also indicated the over representation of Gene Ontology (GO) terms related to endocytosis (Table 1B). The results obtained pointed towards the upregulation of CLTC and other adaptor proteins involved in vesicle coating (Table 1A). CLTC heavy chain (CLTC-Hc) was shown to be upregulated by a fold change of 1.46. The adaptin proteins such as the adaptor-related complexes AP1B1 (1.62 fold change), AP1G1 (1.53 fold change), AP1S3 (2.29 fold change), AP2A1 (1.83 fold change), Synergin (1.26 fold change) and AP3M1 (1.24 fold change) were also upregulated. These are important components of the CLTC-coated vesicles that mediate the internalization of ligand-receptor complexes and the transport of enzymes from the trans-Golgi network (TGN) to lysosomes (Pearse et al. [2000]).

**Table 1:** **Endocytosis related gene expression analysis in the MALME-3M cell line after treatment with albumin-associated lipids**

GENE SYMBOL	GENE DESCRIPTION	ENSEMBL ID	FOLD CHANGE
CLTC	clathrin, heavy chain (Hc)	ENSG00000141367	1.46
TFRC	transferring receptor (p90, CD71)	ENSG00000072274	3.33
AP1B1	adaptor-related protein complex 1, beta 1 subunit	ENSG00000100280	1.62
AP1G1	adaptor-related protein complex 1, gamma 1 subumit	ENSG00000166747	1.53
AP1S3	adaptor-related protein complex 1, sigma 3 subunit	ENSG00000152056	2.29
AP2A1	adaptor-related protein complex 2, alpha 1 subunit	ENSG00000196961	1.83
SYNRG	synergin gamma or AP1 gamma subunit binding protein 1	ENSG00000006114	1.26
AP3M1	adaptor-related protein complex 3, mu 1 subunit	ENSG00000185009	1.24
PICALM	phosphatidylinositol binding clathrin assembly protein	ENSG00000073921	1.20
CLINT1	clathrin interactor 1	ENSG00000113282	2.11
NECAP1	NACAP endocytosis associated 1	ENSG00000089818	1.36
IGF2R	insulin-like growth factor 2 receptor	ENSG00000197081	2.19
M6PR	mannose-6-phosphate receptor (cation dependent)	ENSG00000003056	1.42
PIK3C2B	phosphoinositide-3-kinase, class 2, beta polypeptide	ENSG00000133056	1.79
EEA1	early endosome antigen 1	ENSG00000102189	1.91
LDLR	low density lipoprotein receptor	ENSG00000130164	2.01
LRP1	low-density lipoprotein receptor-related protein	ENSG00000123384	1.70
LDLRAP1	low density lipoprotein receptor adaptor protein	ENSG00000157978	1.29
**ID**	**NAME**	**ADJ p VALUE**	
GO:0016197	endosome transport	1.06E-03	
GO:0005905	coated pit	1.10E-03	
GO:0030139	endocytic vesicle	1.10E-03	
GO:0030131	clathrin adaptor complex	1.25E-03	
GO:0030136	clathrin-coated vesicle	1.25E-03	
GO:0030133	transport vesicle	1.69E-02	
GO:0030135	coated vessicle	1.69E-02	
GO:0030118	clathrin coat	2.09E-02	
GO:0065002	intracellular protein transmembrane transport	2.12E-03	
GO:0042325	regulation of phosphorylation	2.28E-03	
GO:0006897	endocytosis	2.38E-02	
GO:0030666	endocytic vesicle membrane	3.10E-03	
GO:0045121	membrane raft	3.39E-02	
GO:0005768	endosome	3.52E-03	
GO:0032799	low-density lipoprotein receptor metabolic process	3.62E-03	
GO:0001726	ruffle	5.84E-01	
GO:0030117	membrane coat	5.84E-01	

(A) Fold-changes in endocytosis annotated genes. Fold changes were calculated after correcting the number of RNA-seq reads mapped to the gene in each condition by the total number of reads from the sequencing experiment for that condition. (B) Enrichment analysis of GO functional categories related to endocytosis. Adjusted p-values were obtained after Benjamini and Hochberg correction (Benjamini & [Hochberg 1995]). Significant categories (adjusted p-value <0.05) are indicated in bold.

**Figure 3:**
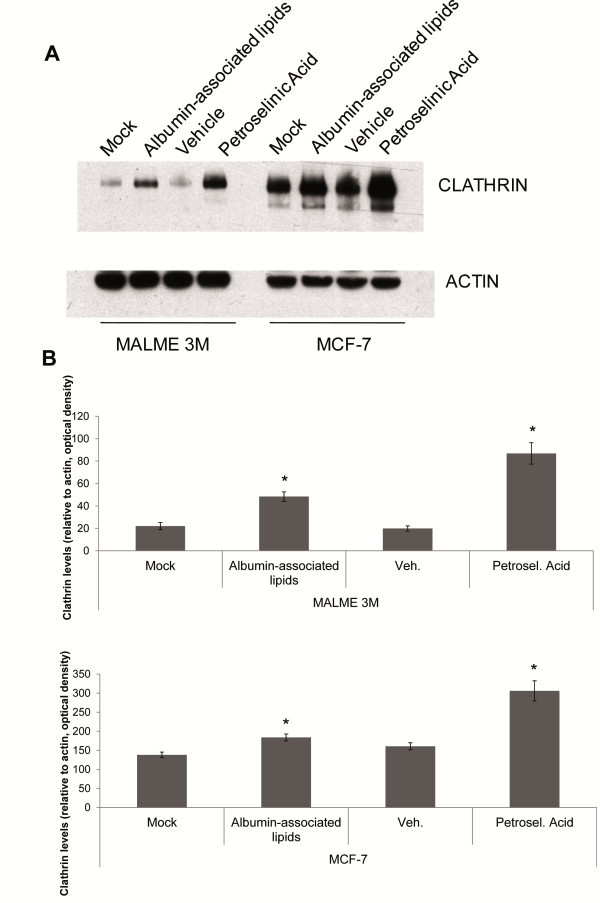
**CLTC expression is increased in the cells treated with albumin-associated lipids and with petroselinic acid.** MALME-3M and MCF-7 cells were treated with albumin-associated lipids and petroselinic acid (100 μg/ml) for 96 hours. Cells were lysed, separated in a 4–12% Bis-Tris gel and probed for western blotting with antibodies against CLTC and Actin (**A**). The western blot band intensity corresponding to CLTC was normalized to the corresponding Actin band for each sample (**B**). The average values from densitometry analyses of three separate experiments are shown. *P <0.05 compared to each control.

As gene expression analysis revealed an upregulation of CLTC and several other adaptin proteins, we tested by western blot analysis if CLTC protein expression was affected. MALME-3M and MCF-7 cell lines were mock-treated, treated with albumin-associated lipids, with a vehicle and with petroselinic acid (100 μg/ml) for 96 hours. Western blot analysis (Figure 3A) and the densitometric analysis of the obtained bands revealed that CLTC protein was significantly increased in MALME-3M and MCF-7 cells in the albumin-associated lipid-treated group compared to the mock-treated group and in the petroselinic acid treated group compared to the vehicle treated group (Figure 3B).

### LD accumulation is impaired by low temperature and by an inhibitor of the CME endocytic pathway

As CLTC expression was increased in MALME-3M and MCF-7 cells as a result of fatty acid co-incubation, we decided to inhibit CME to determine whether this pathway regulated transdifferentiation.

**Figure 4:**
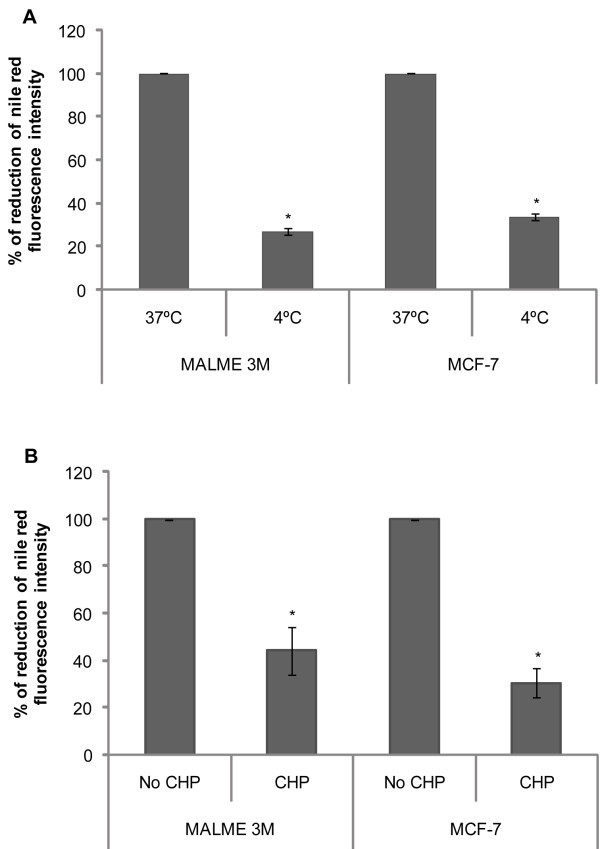
**The CLTC-dependent endocytosis pathway regulates LD accumulation.** Nile Red quantification of neutral lipids in MALME-3M and MCF-7 cells was greatly reduced by low temperatures (**A**) and by CHP (**B**). Cells were kept at 37°C or at 4°C for 120 min. in the presence of petroselinic acid (100 μg/ml) (**A**). Cells were co-incubated for 120 min. with petroselinic acid (100 μg/ml) alone or CHP at a concentration of 50 μg/ml in MALME-3M cells and 100 μg/ml in MCF-7 cells (**B**). The accumulation of neutral lipids was quantified by Nile Red staining in both experiments. The average values from three independent analyses are shown. *P <0.05 compared to each control.

Accumulation of LDs, a known hallmark of adipocyte differentiation (Mersmann et al. [1975]), was quantified by means of the fluorescent dye Nile Red (Greenspan et al. [1985]) and flow cytometry analysis. CME is a process that can be inhibited by low temperature (Sullivan et al. [1976]); to study the effect of temperature in LD accumulation, cells were kept at 37°C or at 4°C for 120 minutes in the presence of petroselinic acid (100 μg/ml). Nile Red fluorescence intensity was drastically reduced in MALME-3M cells by 73±1.62% and in MCF-7 cells by 66±1.56% when compared to their respective controls (Figure 4A). So we concluded that LD accumulation in MALME-3M and MCF-7 cells was a process inhibitable at low temperature.

To further study the effect of CME inhibition on transdifferentiation, cells were pretreated for 30 minutes with chlorpromazine (CHP) (Long et al. [2006]; Inoue et al. [2007]), a drug that disrupts CLTC-mediated endocytosis, and then further incubated with petroselinic acid, in the presence of the inhibitor, for 120 minutes at a concentration of 50 μg/ml in MALME-3M and 100 μg/ml in MCF-7 cells. Nile Red fluorescence intensity was drastically reduced in MALME-3M cells by 56±10.18% and in MCF-7 cells by 70±6.22% when compared to their respective controls (Figure 4B). These studies suggest that LD accumulation is regulated by a CLTC-dependent endocytosis pathway and that inhibition of this pathway attenuates transdifferentiation.

### LD biogenesis is impaired by CLTC siRNA

**Figure 5:**
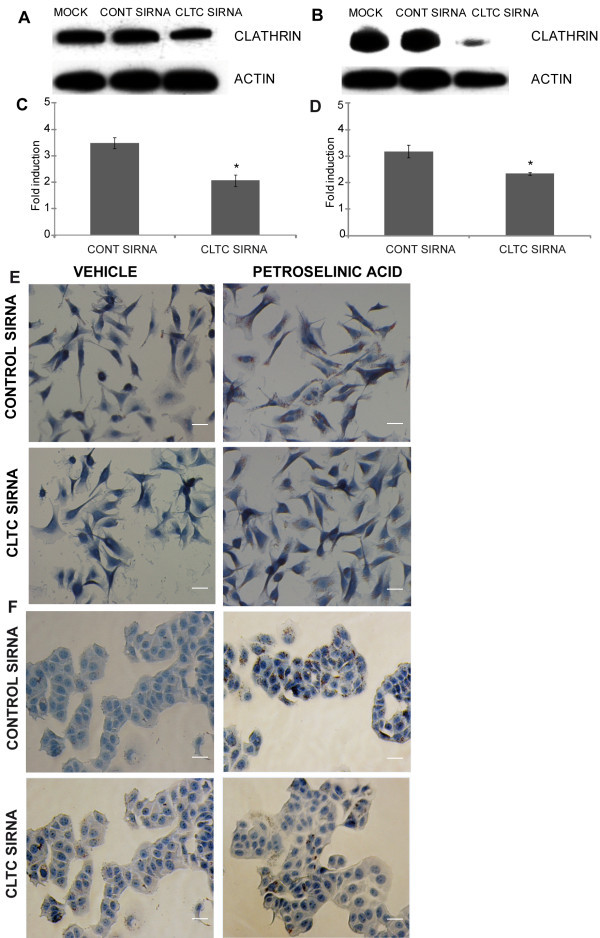
**Inhibition of CLTC levels by SiRNA affects LD biogenesis.** After silencing CLTC expression, biogenesis was examined by Nile Red (**C**, **D**) quantification and Oil Red O semi-quantitative determination (**E**, **F**). Experiments were performed in MALME-3M cells (**C** and **E**) and in MCF-7 cells (**D** and **F**). Cells were transiently transfected with CONT (Control) SiRNA or with CLTC SiRNA for 72 hours prior to the addition of the vehicle 24 hours prior to cell lysation. Cell lysates were then separated in a 4-12% Bis-Tris gel and probed by western blotting with antibodies against CLTC and Actin. The experiment was repeated three times and a representative western blot is shown for MALME-3M cells (**A**) and for MCF-7 cells (**B**). For the neutral lipid determinations, after transfection, cells were treated with either the vehicle or petroselinic acid (100 μg/ml) for 24 hours prior to the testing. Nile Red (**C**, **D**) and Oil Red O and hematoxylin (**E**, **F**) staining were performed as described in Materials and Methods. Quantification was assessed using the FlowJo software. Fold induction was estimated by calculating the ratio between treated conditions (Petroselinic acid) and the untreated condition (vehicle). The average values from three independent analyses are shown. *P <0.05 compared to each control in **C** and **D**. Representative photos are shown in **E** and **F**. The length of the shown size bars is 20 μm.

To further examine dependence of LD biogenesis on a CLTC-mediated pathway, experiments with CLTC-targeting siRNA were conducted (Figure 5). CLTC expression was downregulated by 50% in MALME-3M cells and by 80% in MCF-7 cells compared to control siRNA-transfected cells (Figure 5A and 5B). After transfection and a 24 hour incubation period with petroselinic acid (100 μg/ml), flow cytometry analysis of LD accumulation, quantified by means of the fluorescent dye Nile Red (Greenspan et al. [1985]), showed that CLTC depletion markedly diminished the accumulation of LDs (Figure 5 C and D). The fold change induction by petroselinic acid after CLTC transient silencing was significantly reduced from 3.48 to 2.06 fold in the MALME-3M cells and from 3.15 to 2.15 in the MCF-7 cells.

To corroborate the inhibitory effect of CLTC suppression on the accumulation of LDs, we employed the Oil Red O dye that specifically binds to neutral lipids (Kutt & [Tsaltas 1959]). After transfection and a 24 hour incubation period with petroselinic acid (100 μg/ml), MALME-3M and MCF-7 cells were stained with Oil Red O and hematoxylin (Figure 5E and 5F). The results show a decrease in Oil Red O staining in the CLTC transiently silenced MALME-3M (Figure 5E) and MCF-7 cells, corroborating impairment in neutral lipid accumulation as CLTC is suppressed.

Altogether our results provide strong evidence that CLTC silencing causes a significant change in LD accumulation and in adipocyte-like transdifferentiated MALME-3M and MCF-7 cells.

## Discussion

Clinical cancer therapy, i.e. chemotherapy and radiation, target the actively proliferating tumor cells, which most of the times inevitably induce therapy-resistant cancer cells, derived from proliferative precursor types. Transdifferentiating agents, could avoid chemo- and radio- resistance by shifting the balance from proliferation to differentiation in these tumor precursor types.

In a previous study, albumin-associated lipids were found to induce the transdifferentiation of HCCLs toward adipocyte-like cells (Ruiz-Vela et al. [2011]). Our findings indicated that the connection between albumin-associated lipids and pluripotency (Garcia-Gonzalo & Izpisua [Belmonte 2008]) is far more complex than previously anticipated. In addition to maintaining self-renewal and pluripotency in hESCs (Garcia-Gonzalo & Izpisua [Belmonte 2008]), a role for albumin ( [Kallee 1996]) and its associated lipids (Davis & [Dubos 1947]; Thomas et al. [1995]; Lafond et al. [1994]) has been discovered in adipogenesis in other cell types (Schopfer et al. [2005]). Our results also indicate that certain albumin complex associated poly- and monounsaturated fatty acids induce terminal differentiation and arrest cancer progression (Ruiz-Vela et al. [2011]). Concomitanlty to our studies, Khan et al. have also described the specific growth inhibition of esters of oleic acid and ricinoleic acid against the human skin malignant melanoma cell line (SK-MEL-1) (Khan et al. [2012]). Despite the important findings that we describe here, many key questions remain unanswered in the identification of novel genes and proteins that mediate the terminal transdifferentiation of human cancer cells. To get a better understanding of the mechanisms that lead to transdifferentiation we concentrated our efforts on the role of those genes that are differentially regulated during the process.

EM revealed a marked loss of pigmentation in the melanoma MALME-3M cells treated with albumin-associated lipids, in accordance with the downregulation of MLANA gene expression. Melan-A is known to form a complex with Pmel17 which affects its expression, stability, trafficking, and the processing which is required for melanosome maturation. Its expression is indispensable for Pmel17 function and the formation of cell pigmentation (Hoashi et al. [2005]).

Quantification of gene expression by RNA-seq led to the characterization of the MALME-3M cells treated with albumin-associated lipids. Several adipocytic markers such as PLIN2, LPL and PPARA were significantly upregulated. PPARA could be upregulated merely as a consequence of fatty acid accumulation as it has been shown to be involved in the regulation of obesity in rodents by increasing hepatic fatty acid oxidation (Kersten et al. [1999]). LPL is a well describedadipocyte marker as it regulates the hydrolysis of triglycerides in the adipose tissue (Mead et al. [2002]). Interestingly, PPARG1, an adipogenesis marker shown to be upregulated upon HCCL to adipocyte transdifferentiation (Ruiz-Vela et al. [2011]), showed a differential mapping pattern between the albumin-associated lipid-treated and non-treated cells. The PPARG gene has eight exons which are translated and spliced into different isoforms ( http://www.ensembl.org/Homo_sapiens/Gene/Summary?g=ENSG00000132170;r=3:12328867-12475855), with the PPARG1 isoform being the primary transcript expressed in adipocytes. In treated cells, PPARG showed whole transcript expression, while in mock-treated cells no expression of exons 4 and 5 was evident ( Additional file 3: Supporting information B). These two exons encode the Zinc finger binding site domain of the PPARG1 transcription factor (Finn et al. [2010]). The lack or downregulation of these functional domains in the non-treated cells might be indicative of the differential processing of this gene in the MALME-3M cell lines and of differential expression of the protein (Ruiz-Vela et al. [2011]).

The characterization of the adipocyte-like cells showed that the expression of PLIN2 was increased in MALME-3M, and in MCF-7 cells treated with albumin-associated lipids and with petroselinic acid. PLIN2 is a principal adipocytic marker, which coats lipid droplets in adipocytes (Brasaemle et al. [1997]; Heid et al. [1998]) and its expression has been linked to PPARG1. We previously reported that PPARG1 expression was increased in MALME-3M cells treated with albumin-associated lipids (Ruiz-Vela et al. [2011]). It has been recently reported that pretreatment of murine 3T3-L1 preadipocytes with Rosiglitazone, a potent PPARG1 agonist, decreased lipolysis and increased PLIN2 expression (Kim et al. [2007]).

Quantification of gene expression by RNA-seq also gave us some clues about the possible mechanistic pathways involved in transdifferentiation. Among the genes that were identified as differentially expressed between the treated and the untreated conditions, many of them were related to endocytic functions. Albumin-associated lipid induced transdifferentiation was accompanied by the upregulation of CLTC and other important adaptor-related complexes such as AP1B1, AP1G1, AP1S3, AP2A1, Synergin and AP3M1. Adaptor-related proteins are key components of clathrin coated vesicles that can bind directly to both the clathrin lattice and to the lipid and protein components of membranes (Pearse et al. [2000]). AP1 and AP3 are found at the coated vesicles located at the Golgi complex and it has been suggested that both associate with GLUT4 transporting vesicles and mediate distinct intracellular sorting events at the level of the TGN and endosomes in rat adipocytes (Gillingham et al. [1999]). The observed upregulation of proteins involved in GLUT4 trafficking could be related to the acquisition of the adipocytic phenotype.

Encouraged by the western blot results that showed a clear overexpression of CLTC protein in the transdifferentiated MCF-7 and MALME-3M cells, we decided to determine the role of CME in the adipogenic transdifferentiation process in MALME-3M and in MCF-7 cells. Transient CLTC silencing accomplished by siRNA, caused a significant reduction in LD accumulation induced by petroselinic acid, and further microscopic analysis of Oil Red O and hematoxylin stained cells suggested that not only LD accumulation but also neutral lipid composition in LDs was affected by CLTC silencing.

So far no study has reported the induction of CLTC expression or CME stimulation by monounsaturated fatty acids, although polyunsaturated fatty acids have been found to play a role in the formation of synaptic vesicles and in promoting vesicle budding and membrane trafficking (Darios & [Davletov 2006]; Chernomordik et al. [1997]; Chernomordik et al. [1999]). It was recently reported that α-Synuclein expression, coupled with exposure to physiological levels of polyunsaturated fatty acids, enhanced CLTC mediated endocytosis in neuronal and non-neuronal cultured cells (Ben Gedalya et al. [2009]).

Transferrin receptor (TfR), whose gene expression was upregulated by a fold change of 3.33 in the albumin-associated lipid-treated MALME-3M cells (Table 1A), is internalized from the cell plasma membrane and recycled back to the cell plasma membrane specifically via CME (Hanover et al. [1984]). El-Jack et al. reported that murine 3T3-L1 preadipocytes, in a differentiation media previously described (Stephens et al. [1997]), showed a gradual increase in whole cell TfR levels but a decrease in cell surface TfR levels (El-Jack et al. [1999]). The results obtained in this study indicated that the differentiation process might account for the observed alterations in internalization and/or TfR recycling. These results may be useful in understanding why CME is of critical importance in HCCL to adipocyte transdifferentiation.

The clarification of the roles played by the differentially expressed genes and proteins in the process of adipogenic transdifferentiation in HCCL cultures should provide the basic foundation to develop novel molecules for cancer therapy.

## Electronic supplementary material

Additional file 1: **Supporting information A.** Gene expression in the endocytic pathway and at the PPARG locus after treatment with albumin-associated lipids. (**A**) Downregulated and upregulated genes after treatment with albumin-associated lipids are colored in blue and red, respectively. Intensity is proportional to the log_2_ of the ratio between the two conditions. Significantly differentially expressed genes are indicated by bold box lines. (TIFF 242 KB)

Additional file 2: **Supporting information B.** Gene expression in the endocytic pathway and at the PPARG locus after treatment with albumin-associated lipids. (**B)** Mapped reads of gene expression at the PPARG locus are shown piled up on mapping positions. Expression at exons 4 and 5 in the PPARG1 transcript was observed only after albumin incubation. (TIFF 80 KB)

Additional file 3: **Supporting information C.** Electron Microscopy at high magnification of melanosome pigmentation in MALME-3M cells mock-treated or treated with Albumin-associated lipids. MALME-3M cells were mock-treated or treated with albumin-associated lipids for 24 hours. The arrows indicate the dark vesicles that are hallmarks of melanosomes and disappear upon albumin-associated lipid treatment. (TIFF 1043 kb) (TIFF 1 MB)
