# Supplementary figures and images for: Transdifferentiation of MALME-3M and MCF-7 Cells toward Adipocyte-like Cells is Dependent on Clathrin-mediated Endocytosis
Source: Springerplus. 2012 Oct 30;1:44. doi: 10.1186/2193-1801-1-44 (PMC3725915; doi:10.1186/2193-1801-1-44)

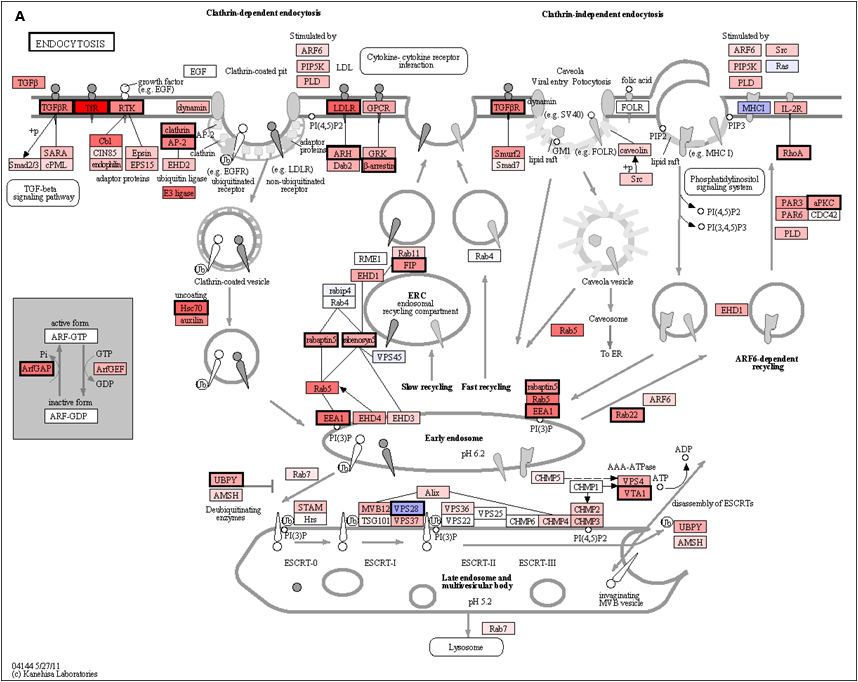

Supplement: Supplementary file 1 — Additional file 1: Supporting information A. Gene expression in the endocytic pathway and at the PPARG locus after treatment with albumin-associated lipids. (A) Downregulated and upregulated genes after treatment with albumin-associated lipids are colored in blue and red, respectively. Intensity is proportional to the log2 of the ratio between the two conditions. Significantly differentially expressed genes are indicated by bold box lines. (TIFF 242 KB) [file 40064_2012_36_MOESM1_ESM.tiff]

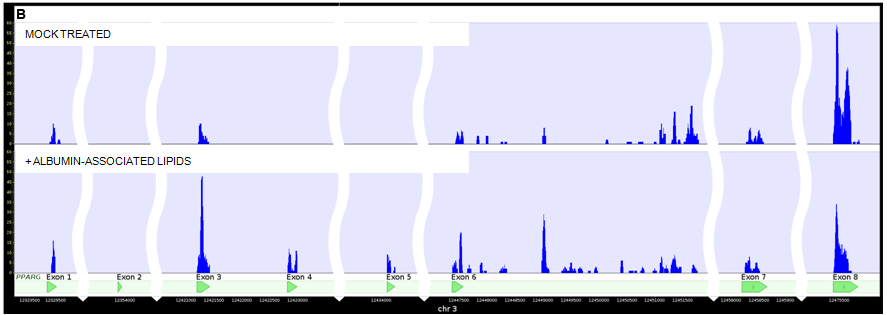

Supplement: Supplementary file 2 — Additional file 2: Supporting information B. Gene expression in the endocytic pathway and at the PPARG locus after treatment with albumin-associated lipids. (B) Mapped reads of gene expression at the PPARG locus are shown piled up on mapping positions. Expression at exons 4 and 5 in the PPARG1 transcript was observed only after albumin incubation. (TIFF 80 KB) [file 40064_2012_36_MOESM2_ESM.tiff]

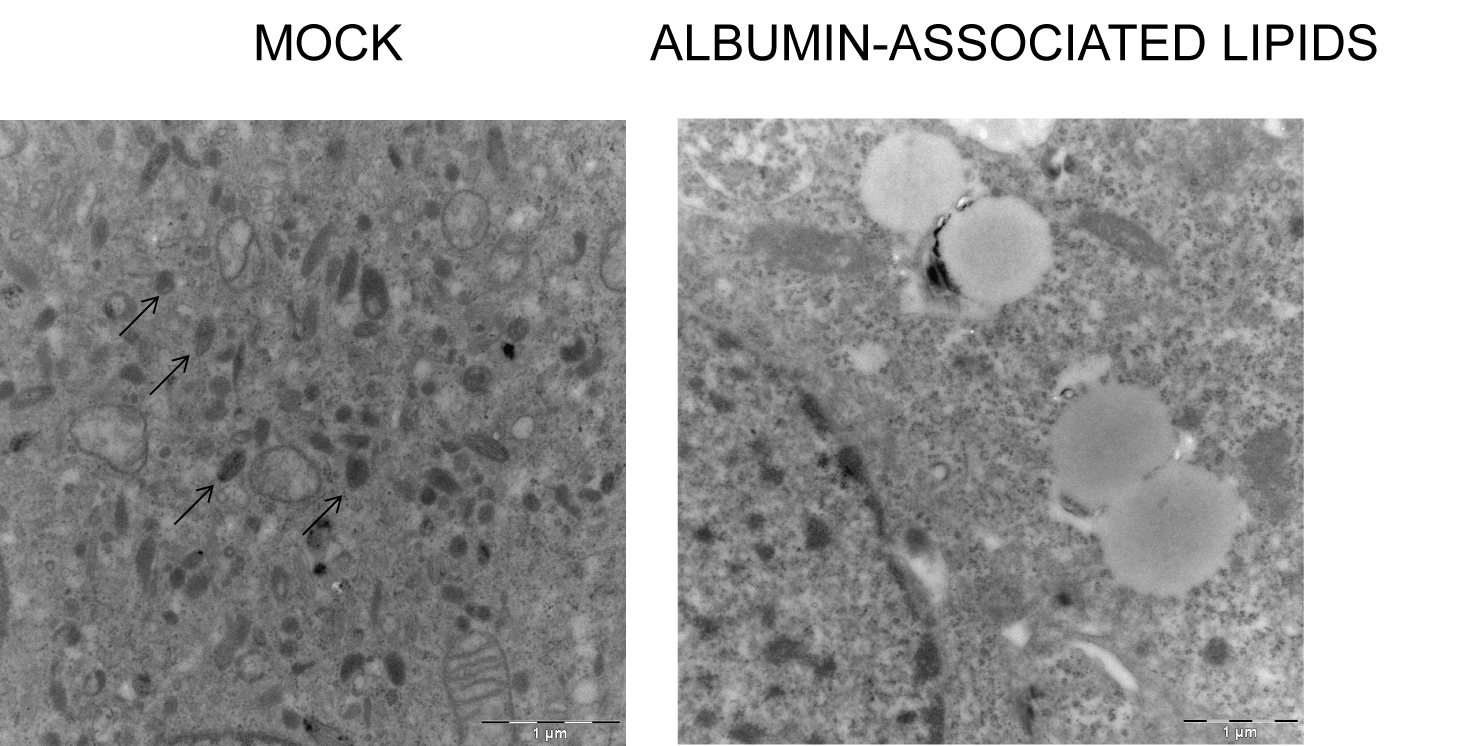

Supplement: Supplementary file 3 — Additional file 3: Supporting information C. Electron Microscopy at high magnification of melanosome pigmentation in MALME-3M cells mock-treated or treated with Albumin-associated lipids. MALME-3M cells were mock-treated or treated with albumin-associated lipids for 24 hours. The arrows indicate the dark vesicles that are hallmarks of melanosomes and disappear upon albumin-associated lipid treatment. (TIFF 1043 kb) (TIFF 1 MB) [file 40064_2012_36_MOESM3_ESM.tiff]
